# Focus on the cetirizine use in clinical practice: a reappraisal 30 years later

**DOI:** 10.1186/s40248-019-0203-6

**Published:** 2019-12-06

**Authors:** Angelo G. Corsico, Salvatore Leonardi, Amelia Licari, Gianluigi Marseglia, Michele Miraglia del Giudice, Diego G. Peroni, Carmelo Salpietro, Giorgio Ciprandi

**Affiliations:** 1 Division of Respiratory Diseases, Foundation IRCCS Policlinico San Matteo, University of Pavia, Pavia, Italy; 2 Department of Clinical and Experimental Medicine, University of Catania, Catania, Italy; 3 Department of Pediatrics, Foundation IRCCS Policlinico San Matteo, University of Pavia, Pavia, Italy; 4 Department of Woman, Child and of General and Specialized Surgery, University of Campania “Luigi Vanvitelli”, Naples, Italy; 5 U.O. Pediatria, Azienda Ospedaliero-Universitaria Pisana, Scuola di Specializzazione in Pediatria, University of Pisa, Pisa, Italy; 6 Department of Pediatrics, Unit of Pediatric Genetics and Immunology, University of Messina, Messina, Italy; 7 Allergy Clinic, Casa di Cura Villa Montallegro, Genoa, Italy

**Keywords:** Antihistamines, Cetirizine, Clinical practice, Histamine, H1 receptors

## Abstract

Antihistamines are currently one of the most commonly administered categories of drugs. They are used to treat symptoms that are secondary to histamine release, which is typical of certain allergic conditions, including rhinitis, conjunctivitis, asthma, urticaria, and anaphylaxis. Cetirizine belongs to the second-generation family, so, it is very selective for peripheral H1 receptors, is potent and quickly relieves symptoms, exerts additional anti-allergic/anti-inflammatory effects, and is usually well-tolerated. It has been marketed 30 years ago. In these years, a remarkable body of evidence has been built. The current review provides a practical update on the use of cetirizine in clinical practice.

## Background

Histamine is the pivotal mediator of an allergic reaction. The concentration of histamine is particularly high in mast cells, that reside in the respiratory tree, gastrointestinal tract, and skin. Histamine can act on four types of receptors: H1, H2, H3, and H4. H1 and H2 receptors are distributed in both the peripheral and the central nervous system (CNS) and allow histamine to exert effects on smooth muscles and glands. By acting on H1, histamine causes itching, stimulates secretion from the nasal mucosa, contracts smooth muscle in the bronchi and intestines, and relaxes smooth muscle in small blood vessels. Additionally, histamine stimulates gastric acid secretion via H2 receptors. H3 receptors are mainly expressed in the CNS and act as an autoreceptor on histaminergic neurons, inhibiting the release of histamine and modulating that of other neurotransmitters. H4 receptors are found on cells of the immune system, in the gastrointestinal tract, in the CNS, and on afferent neurons with primary sensors. The action of histamine on H4 receptors induces chemotaxis, cytokine secretion, and upregulation of adhesion molecules [1].

H1-antihistamines are widely used in patients to treat symptoms that are secondary to histamine release, which is typical of certain allergic conditions. It is possible to distinguish first and second-generation antihistamines; pharmacological effects and therapeutic applications are similar, but second-generation antihistamines have fewer adverse effects because they are more selective for peripheral H1 receptors [1].

Some second-generation drugs have also some important anti-inflammatory and anti-allergic effects that occur by a decrease in : 1) production of cytokines by proinflammatory drugs and in the release of other mediators by mastocytes and basophils; 2) recruitment of eosinophils in the late phase of allergic reactions; 3) expression of membrane receptors in nasal epithelial cells and the vascular endothelium, particularly the leukocyte Intercellular Adhesion Molecule 1 (ICAM-1), which favours leukocyte migration from the blood to the respiratory mucosa and constitutes the main receptor for respiratory viruses to which the untreated atopic subject appears to be more susceptible [2].

Cetirizine is a second-generation antihistamine and was launched in the Italian market in 1989, such as 30 years ago. In these years, cetirizine was the most relevant compound in its class and is still very popular. However, at the beginning of the twenty-first century, levocetirizine was commercialized worldwide for a patent reason. Therefore, most of the studies were conducted investigating levocetirizine, without demonstrating a superiority, nor in efficacy or in safety, of the enantiomer compared to the racemic compund. Nevertheless, some studies have been performed still investigating cetirizine. The current review reports a summary of the well-known literature and updates the most recent information on cetirizine.

### Pharmacological characteristics

Cetirizine hydrochloride (chlorophenyl-phenylmethyl-piperazinyl ethoxy-acetic acid) is a racemic mixture composed of equal amounts of two enantiomers, levocetirizine, and dextrocetirizine, which do not undergo interconversion and therefore remains stable [3]. Cetirizine belongs to the piperazine family.

### Clinical indications

The therapeutic indications of cetirizine are the treatment of nasal and ocular symptoms in seasonal and perennial allergic rhinitis and the treatment of chronic idiopathic urticaria. It is available as a 10 mg tablet. The standard dosage is 10 mg once a day.

There is a large quantity of controlled randomized trials conducted in these two diseases. Different exhaustive reviews have been published in the past years [4–6]. Here, the literature concerning the pharmacological characteristics and the clinical efficacy of cetirizine in adults is summarized and updated [7].

#### Pharmacodynamic profile

Antihistaminic activity: cetirizine is a very selective H1 receptor antagonist. Cetirizine has a value of concentration producing 50% inhibition of H1 receptors of 0.65 μmol/L, but very low affinity for other receptors, including α1-adrenergic, D2 dopaminergic, 5-HT2 serotoninergic, and muscarinic, such as > 10 μmol/L [8]. Oral cetirizine 10 mg is more effective than other antihistamines in inhibiting the histamine-induced wheal and flare [9]. The increase in nasal resistance after histamine nasal challenge is better protected by cetirizine than loratadine [10].

Antiallergic and anti-inflammatory activity: using allergen or non-specific challenges, several studies demonstrated the capability of cetirizine in reducing the cellular infiltration and the expression of adhesion molecules [11].

Effects on the central nervous system: cetirizine is without significant CNS activity, binds to about 30% of H1 cerebral receptors; several studies investigated this topic and reported no clinically relevant side effects [12].

Effects on the cardiovascular system: cetirizine had no clinically relevant effect on the QT or QT_c_ interval (even at 60 mg/day for a week); the combination with agent metabolized by cytochrome P450 does not affect plasmatic concentration [13].

#### Pharmacokinetic profile

Cetirizine is a zwitterion, with high binding to mainly serum albumin and low apparent volume of distribution, as well as low brain uptake [14], which are indicative of a low affinity for lean tissues such as the myocardium (thus providing low cardiotoxicity) and low/lack of sedative effects, respectively. It has been demonstrated that cetirizine has a low volume of distribution, therefore it reaches the target organs at an effective concentration. Cetirizine is absorbed extensively and rapidly from the gut [15], leading to high bioavailability and rapid onset of action [16]. Unlike many other second-generation antihistamines, cetirizine does not undergo hepatic metabolism to any appreciable extent but is excreted mostly unchanged in the urine, both in healthy volunteers and patients with chronic liver disease [17]. The lack of hepatic metabolism entails a low potential for drug-drug interactions, avoiding any toxic effects with drugs subjected to metabolism by P450 enzymes and transmembrane transport [18]. Cetirizine has an elimination half-life of around 10.5 h in healthy volunteers [19], therefore, it may be used once daily. Cetirizine has a high affinity and selectivity for the H1 receptor, consequently, it has a more potent, faster onset, and prolonged action than other antihistamines [20]. In elderly subjects, the mean serum concentration correlates with creatinine clearance than with age per se [21].

### Therapeutic efficacy

There are several clinical trials providing evidence of cetirizine efficacy in patients with seasonal allergic rhinitis (SAR), perennial allergic rhinitis (PAR), chronic spontaneous urticaria (CSU), atopic dermatitis, and allergic asthma.

#### Allergic rhinoconjunctivitis

A review performed a comprehensive literature search for publications concerning the clinical use of cetirizine in patients, both adults and children, with allergic rhinitis [6]. This review is exhaustive and complete but is updated on February 2013. Actually, from that date, some studies have been published.

Skoner and colleagues evaluated the effect of cetirizine on symptom severity and health-related quality of life (QOL), using a disease-specific instrument, in adults with PAR [22]. The study was randomized, double-blind, and placebo-controlled. It was conducted at 15 U.S. centres outside the pollen allergy season. After a 1-week placebo run-in period, qualified adult PAR patients were randomized to once-daily cetirizine 10 mg (*n* = 158) or placebo (*n* = 163) for 4 weeks. Change from baseline in total symptom severity complex (TSSC) and overall Rhinitis QOL Questionnaire (RQLQ) scores were the primary outcomes. Cetirizine significantly improved the mean TSSC for each treatment week (*p* < 0.05) and for the entire period (*p* = 0.005) in comparison with the placebo. After 4 weeks, cetirizine-treated subjects reported a significantly greater overall improvement in RQLQ scores compared with placebo-treated subjects (*p* = 0.004). After 1 week, cetirizine produced significant improvements in the nasal symptoms, practical problems, and activities RQLQ domain scores compared with placebo (*p* < 0.05). At the end of treatment, cetirizine-treated subjects reported significant reductions in these RQLQ domain scores and emotion domain scores compared with placebo-treated subjects (*p* < 0.05).

Urdaneta reported the effects of different cetirizine dosing schedules in comparison to twice daily (BID) chlorpheniramine and placebo on SAR symptoms at 12 and 24 h post-dose [23]. The first study included subjects who received cetirizine 10-mg once daily in the morning (QAM), cetirizine 10-mg once daily at bedtime (QHS), cetirizine 5-mg twice daily, or placebo. The second study evaluated subjects who received cetirizine 5-mg QAM, cetirizine 10-mg QHS, chlorpheniramine 8-mg BID, or placebo. The primary outcome was the total symptom severity complex (TSSC). *Post-hoc *analyses of reflective symptom severity assessed in the morning (TSSCAM) and the evening (TSSCPM) were conducted to evaluate cetirizine’s effects at 12 and 24 h post-dose. The first study showed that subject- and investigator-assessed TSSC was significantly lower in all cetirizine groups versus placebo (*p* < 0.003). The second study demonstrated that subject-assessed TSSC was significantly lower in all cetirizine groups versus placebo (*p* < 0.04) and was numerically lower for investigator-assessed TSSC. *Post-hoc *analyses demonstrated that cetirizine significantly improved TSSCAM at 12 and 24 h post-dose versus placebo in both studies regardless of the dosing schedule. Therefore, regardless of the dosing regimen, cetirizine demonstrated effective 24-h relief of SAR symptoms, mainly on TSSCAM, which reflects overnight and early morning symptom control.

Two recent studies were performed using a topical formulation, such as cetirizine ophthalmic solution 0.24%. The first published trial evaluated the efficacy and safety of cetirizine ophthalmic solution 0.24% compared with vehicle in the treatment of allergen-induced conjunctivitis using the conjunctival allergen challenge (CAC) model [24]. The trial included a single-centre (Study 1) and a multi-centre (Study 2), double-masked, randomized, vehicle-controlled, parallel-group. CAC studies were conducted over ~ 5 weeks and four study visits. The study design differed in entry criteria: Study 2 required more severe allergic conjunctivitis symptoms. Approximately 100 subjects were randomized in each study. Ocular itching and conjunctival redness 15 min and 8 h post-treatment, post-CAC, were the primary outcomes. Cetirizine treatment administered 15 min or 8 h before CAC resulted in significantly lower ocular itching at all time points post-CAC (*p* < 0.0001) compared to vehicle in both studies. Conjunctival redness measured by the investigator was significantly lower after cetirizine treatment compared to vehicle at 7 min post-CAC at both 15 min and 8 h post-treatment in both studies (*p* < 0.05). All secondary endpoints were in favour and confirmatory of cetirizine efficacy with significant improvement in chemosis, eyelid swelling, tearing, ciliary redness, and episcleral redness, as well as nasal symptoms (rhinorrhea, nasal pruritus, ear or palatal pruritus, and nasal congestion) post-CAC. Cetirizine ophthalmic solution 0.24% was well tolerated in both studies. The second published trial aimed to assess the safety and tolerability of cetirizine ophthalmic solution 0.24% for the treatment of ocular itching associated with allergic conjunctivitis [25]. Three different clinical studies evaluated cetirizine ophthalmic solution 0.24% administration: a Phase I prospective, single-center, open-label, pharmacokinetic (PK) study (*N* = 11 subjects) evaluating single-dose administration and BID administration for 1 week in healthy adults, and two Phase III, multi-center, randomized, double-masked, vehicle-controlled, parallel-group studies evaluating the safety and tolerability in adult and pediatric populations (2–18 years of age) for up to 6 consecutive weeks. The first safety and tolerability study evaluated cetirizine BID (study 1, *N* = 512 subjects), while the second study examined cetirizine three times daily (study 2, *N* = 516 subjects). Each study assessed best-corrected visual acuity, slit-lamp biomicroscopy, intraocular pressure, dilated ophthalmoscopy, treatment-emergent adverse events, vital signs, urine pregnancy tests, and physical examination (general health, head, eyes, ears, nose, and throat). The PK study also measured haematology, blood chemistry, and urinalysis, while the two Phase III studies additionally assessed corneal endothelial cell counts (ECC) and ECC density in a subset of subjects (via specular microscopy), and drug administration tolerability. Bilateral administration of cetirizine ophthalmic solution 0.24% resulted in low systemic exposure in the PK study and was associated with a low incidence of mild adverse events. There were no drug-related severe or serious adverse events. The tolerability scores between the active and vehicle groups were comparable, demonstrating high comfort in the administration of cetirizine ophthalmic solution 0.24%.

#### Chronic spontaneous urticaria

A recent Cochrane meta-analysis evaluated H1-antihistamines for CSU [26]. This review confirmed the efficacy of cetirizine in the treatment of patients suffering from CSU. Actually, from that date, some other studies have been published.

Guevara-Gutierrez and colleagues conducted a randomized, double-blind, placebo-controlled trial, including 32 patients with chronic urticaria [27]. Group A (16 patients) was treated with cetirizine plus ranitidine; Group B (16 patients) was treated with cetirizine plus placebo, both for 30 days. Efficacy measures were Urticaria Activity Score (UAS), Chronic Urticaria QOL Questionnaire (CU-Q2oL) and time of symptom remission, safety measures were clinical and laboratory effects. Complete remission was obtained in 10 patients (62.5%) from Group A and 7 patients (44%) from Group B (*p* = 0.28). The UAS in Group A was 1.53 ± 2.09 versus Group B 2.06 ± 1.34 (*p* = 0.20). The CU-Q2oL in Group A was 12.93 ± 19.20 versus Group B 12.68 ± 10.30 (*p* = 0.20). At the end of treatment, 13 patients (81%) from Group A and 14 patients (87.5%) from Group B had some type of adverse effect (*p* = 1.0). Therefore, the authors concluded that the combination of cetirizine with ranitidine was not more effective than cetirizine alone in chronic urticaria.

A multicenter, triple-blind, randomized study investigated the change in a histamine-induced wheal and flare measurements 24 hours after administration of antihistamines, including cetirizine, to predict the efficacy of treatment [28]. Patients received a daily oral dose of cetirizine, fexofenadine, bilastine, desloratadine, or ebastine over 8 weeks. After 4 weeks, a higher dose of antihistamine was administered to patients who did not experience a clinical response. A histamine skin prick test was carried out at baseline and 24 hours after the first dose of antihistamine. Disease severity as assessed by UAS, response to the histamine skin prick test, and impact on the patient’s quality of life (Dermatology Life Quality Index [DLQI]) were assessed every 2 weeks. The study population consisted of 150 patients (30 per group) and 30 controls. Twenty-four hours after administration of antihistamine, inhibition of the histamine wheal by > 75% was significantly associated with better UAS and DLQI scores. The safety and efficacy of the 5 antihistamines were similar. After up-dosing, rates of disease control (DLQI score < 5) increased from 58.7 to 76.7%. Cetirizine was able to significantly affect the cutaneous response.

#### Unconventional use

Antihistamines may have a role in patients suffering from allergic asthma associated with allergic rhinitis. In fact, during allergic rhinitis and asthma, the upper and lower airways are affected by a common inflammatory process that can be sustained and amplified by interconnected mechanisms [29]. Allergic rhinitis and non-specific vasomotor rhinitis are some of the most important risk factors for the onset of asthmatic disease, and they are therefore important aggravating factors, as promote the airway inflammation diffusion to the bronchi [30, 31]. Thus, therapy with anti-H1 antihistamines could confer an additional benefit in the control of asthmatic symptoms in subjects with concomitant allergic rhinitis and bronchial asthma [32]. In this regard, some studies evaluated the efficacy of cetirizine in patients with mild or moderate asthma and associated allergic rhinitis [33–37]. These studies, randomized and placebo-controlled, showed that doses ranging from 10 to 30 mg of cetirizine determined an improvement in asthma symptoms (but not always in pulmonary function tests), especially when the treatment reached 5–6 weeks [33–37]. A key mechanism of action on resident cells involved the cell trafficking and bronchial inflammation downregulating the adhesion molecules machinery, namely intracellular adhesion molecule 1, after long-term cetirizine administration [38].

Therefore, even though the cetirizine use in asthma remains off label, patients with allergic rhinitis and concomitant asthma can be favourably treated with cetirizine in clinical practice.

#### Safety issue

Several studies investigating the safety profile of cetirizine have been conducted in these last years. Du and Zhou performed a meta-analysis concerning the somnolence effect of cetirizine [39]. The review aimed to assess the somnolence effect of cetirizine 10 mg daily compared to placebo in patients aged 6 years and older using meta-analysis and explore the sources of heterogeneity among different studies. Databases were searched for randomized controlled trials (RCTs) of cetirizine. Overall risk differences (RDs) were determined by meta-analyses of 13 trials using the DerSimonian and Laird method based on fixed-effects and random-effect models, respectively. The Q statistic, H statistic, and I(2) were calculated for heterogeneity analysis. Subgroup analysis, Galbraith plot, sensitivity analysis, and meta-regression were also performed to explore the sources of heterogeneity. Various analyses showed that heterogeneity existed among the 13 trials and the placebo run-in period was the cause of heterogeneity. For RCTs without and with placebo run-in period, the overall RDs were 6.51% (95% CI, 4.47 to 8.56%) and 1.03% (95% CI, − 0.13 to 2.19%), respectively, showing that the difference in somnolence rate between cetirizine 10 mg daily and placebo was not statistically significant for the subgroup with placebo run-in. Therefore, this meta-analysis suggested that cetirizine 10 mg daily has no somnolence effect compared to placebo.

Pasko and colleagues conducted a systematic review to investigate the importance of drug-food interaction for second-generation H1-antihistamine drugs [40]. Systematic literature queries were performed in Medline (via PubMed), Cochrane Library, Embase, and Web of Science. The queries covered nine specific names of second-generation antihistamines, namely bilastine, cetirizine, desloratadine, ebastine, fexofenadine, levocetirizine, loratadine, mizolastine, and rupatadine, in combinations with such terms as “food”, “juice”, “grapefruit”, “fruits”, “alcohol”, “pharmacokinetics”, and “meal”. Additional publications were found by checking all the reference lists. Where none data on drug-food interaction could be found within the investigated databases, a specific drug prescribing information was used. Globally, 2326 publications were identified with the database queries. Articles were subjected to analysis by reviewing their title, abstract and full text; duplicated papers were removed. Having collected a complete set of data, a critical review was undertaken. For selected H1-antihistamines food, fruit juices or alcohol consumption may significantly impact the efficacy and safety of the therapy, even though cetirizine was more relevant alcohol intake. This issue shall be well understood to educate patients properly, as it provides the major therapeutic element in allergic diseases.

An international panel of experts evaluated the risk of ventricular tachyarrhythmia (VA) related to the use of individual antihistamines [41]. A matched case-control study nested in a cohort of new users of antihistamines was conducted within the EU-funded ARITMO project. Data on 1997–2010 were retrieved from seven healthcare databases: AARHUS (Denmark), GEPARD (Germany), HSD and ERD (Italy), PHARMO and IPCI (Netherlands) and THIN (UK). Cases of VA were selected and up to 100 controls were matched to each case. The odds ratio (OR) of current use for individual antihistamines was estimated using conditional logistic regression. For cetirizine and levocetirizine, no VA risk was found. A statistically significant, increased risk of VA was found only for current use of cyclizine in the pooled analysis (ORadj, 5.3; 3.6–7.6) and in THIN (ORadj, 5.3; 95% CI, 3.7–7.6), for dimetindene in GEPARD (ORadj, 3.9; 1.1–14.7) and for ebastine in GEPARD (ORadj, 3.3; 1.1–10.8) and PHARMO (ORadj, 4.6; 1.3–16.2). Therefore, the risk of VA associated with a few specific antihistamines could be ascribable to heterogeneity in the pattern of use or receptor binding profile.

Another study collected information on the pregnancy outcomes of women exposed to the antihistamine cetirizine [42]. The UCB Pharma Patient Safety Database was searched for pregnancies up to 28 February 2015. The objective of this study was to provide maternal cetirizine exposure reports; pregnancy outcomes were examined, including exposure, comorbidities, and infant events. Outcomes were available for 228 of 522 pregnancies with maternal cetirizine exposure; 49 were prospective. The majority (83.7%) resulted in live births; four spontaneous miscarriages, three induced abortions and one stillbirth were reported. Most pregnancies were exposed during the first trimester. Two congenital malformations were reported. The results suggest that cetirizine exposure was not associated with adverse pregnancy outcomes above the background rates. While reassuring, the strengths and limitations of a safety database study need to be considered. Therefore, this study suggests that cetirizine exposure during pregnancy is not linked to an increase in adverse outcomes. Cetirizine exposure mainly happened during the first trimester only, when most organogenesis takes place.

Furthermore, four cases of hepatotoxicity due to cetirizine use were reported in patients without a history of alcohol use, blood transfusion, tooth extraction, any surgical operation, close contact with hepatitis patients, history of a systemic disease or any other drug use [43]. Cetirizine was therefore considered to the likely cause for the increased values of the liver tests (AST, ALT, ALP, GGT and total bilirubin). The authors concluded that in patients with high levels of liver enzymes of unknown origin, cetirizine, as well as other hepatotoxic drugs, should be reconsidered.

## Conclusions

Cetirizine is a potent second-generation H1 antihistamine. It is clinically efficacious in allergic rhinitis and chronic spontaneous urticaria. Cetirizine improves quality of life and reduces symptom severity. Cetirizine has a rapid onset of action and long half-life that allows once-daily dosing. Cetirizine is excreted by the kidney. Its use is safe and well-tolerated, even though the most common side effects are mild somnolence and dry mouth, both of them dose-dependent. Cetirizine has also an antiallergic and anti-inflammatory activity that could be fruitfully used in clinical practice. Therefore, cetirizine is, also after 30 years, a first-choice antihistamine.

## Data Availability

Not applicable.

## Abbreviations

(TSSCAM): TSSC assessed in the morning
(TSSCPM): TSSC assessed in the evening
BID: Twice daily
CAC: Conjunctival allergen challenge
CNS: Central nervous system
CSU: Chronic spontaneous urticaria
CU-Q2oL: Chronic urticaria QOL questionnaire
DLQI: Dermatology life quality Index
ECC: Endothelial cell counts
ICAM-1: Intercellular adhesion molecule 1
OR: Odds ratio
PAR: Perennial allergic rhinitis
PK: Pharmacokinetic
QAM: Once daily in the morning
QHS: Once daily at bedtime
QOL: Quality of life
RCTs: Randomized controlled trials
RDs: Risk differences
RQLQ: Rhinitis QOL questionnaire
SAR: Seasonal allergic rhinitis
TSSC: Total symptom severity complex
UAS: Urticaria Activity Score
VA: Ventricular tachyarrhythmia
